# Dysentery

**Published:** 1856-10

**Authors:** 


*Dysentery.*

Dr. Shoemaker, of Columbia, Monroe County, states, in a recent letter to us, that dysentery has been very prevalent and severe in that locality during the last part of summer and autumn. We have received similar intelligence from several other neighborhood, including places in Michigan, Wisconsin, and Iowa.

In this city, during the months of July and August, many children under two years of age suffered from attacks of this disease; and during the last month (September) cases of typhoid and typhus fevers have been more frequent than usual. Still, the ratio of mortality, from all diseases, during the year, has been low, and the season will be ranked as one of good health. A more full report in regard to the diseases of the past summer and autumn, noting their peculiarities, both pathological and therapeutical, will be published in our next number.

